# Additive effect of in-hospital TIMI bleeding and chronic kidney disease on 1-year cardiovascular events in patients with acute coronary syndrome: Data from Taiwan Acute Coronary Syndrome Full Spectrum Registry

**DOI:** 10.1007/s00380-014-0504-9

**Published:** 2014-05-07

**Authors:** Tsung-Hsien Lin, Wen-Ter Lai, Chi-Tai Kuo, Juey-Jen Hwang, Fu-Tien Chiang, Shu-Chen Chang, Chee-Jen Chang

**Affiliations:** 1 Division of Cardiology, Department of Internal Medicine, Kaohsiung Medical University Hospital, No.100 Tzyou 1st Road, Kaohsiung, 80708 Taiwan, ROC; 2 Department of Internal Medicine, Faculty of Medicine, Kaohsiung Medical University, Kaohsiung, Taiwan; 3 Chang Gung University College of Medicine, Taoyuan, Taiwan; 4 Division of Cardiology, Department of Internal Medicine, Linkou Chang Gung Memorial Hospital, Linkou, Taiwan; 5 Division of Cardiology, Department of Internal Medicine, National Taiwan University Hospital, Taipei, Taiwan; 6 Division of Biostatistics, Institute of Public Health, National Yang-Ming University, Taipei, Taiwan; 7 Graduate Institute of Clinical Medicine, Research Center for Clinical Informatics and Medical Statistics, Chang Gung University, Taoyuan, Taiwan

**Keywords:** Acute coronary syndrome, Chronic kidney disease, Bleeding

## Abstract

In-hospital bleeding (IHB) is associated with the risk of subsequent cardiovascular events (CVE) in acute coronary syndrome (ACS). We investigated whether increased risk of CVE by IHB is influenced by chronic kidney disease (CKD) or both have detrimental effects on CVE. In a Taiwan national-wide registry, 2819 ACS patients were enrolled. CKD is defined as an estimated glomerular filtration rate of <60 ml/min per 1.73 m^2^. The primary end point is the composite of death, non-fatal myocardial infarction and non-fatal stroke at 12 months. 53 (1.88 %) and 949 (33.7 %) patients suffered from IHB and CKD, respectively. Both IHB and CKD are independently associated with increased risk of the primary end point (HR 2.04, 95 % CI 1.05–3.99, *p* = 0.037 and HR 2.17, 95 % CI 1.63–2.87, *p* < 0.01, respectively). The Kaplan–Meier curves show significantly higher event rates among those with IHB and CKD in the whole, ST-elevation and non-ST elevation populations (all *p* < 0.01). Patients with IHB(+)/CKD(−), IHB(−)/CKD(+) and IHB(+)/CKD(+) have 1.88-, 2.13- and 2.98-fold risk to suffer from the primary end point compared with those without IHB and CKD (*p* = 0.23, <0.01 and <0.01, respectively). IHB or CKD is independently associated with poor cardiovascular outcome and patients with both IHB and CKD have the worst outcome in ACS.

## Introduction

Cardiovascular disease (CVD) accounts for approximately one-third of all global deaths [1]. The prevalence of CVD has increased considerably in Asian countries over the past several decades as a result of shifts toward a more “westernized” lifestyle. In Taiwan, CVD is the second most common cause of mortality since 2010 [2]. Acute coronary syndrome (ACS) is the most severe form of CVD. Because of its major impact on morbidity and mortality, as well as its contribution to annual health-care costs, it is of the utmost importance to develop improved strategies for reducing cardiovascular events (CVE) and preventing complications.

In ACS, aggressive antiplatelet and anticoagulation therapies have been recently developed and can reduce future CVE, but may increase the risk of bleeding. Anemia and bleeding events have been shown to increase mortality in studies of ACS and percutaneous coronary intervention (PCI) [3, 4]. Because lower body weight could be associated with bleeding complication in ACS, weight-adjusted dose of antithrombotic agent is recommended in the international ACS guidelines [5, 6]. Compared with Caucasians, the Asian population usually has lower body weight and might possibly suffer from antithrombotic and antiplatelet overdose. Although bleeding events increase the risk of mortality in the Caucasian population, no study has been reported in the Asian population.

Chronic kidney disease (CKD) is a risk factor for coronary heart disease and bleeding with antithrombotic therapy in patients with ACS [7, 8]. Whether the association between bleeding and mortality is influenced by the presence of CKD or both have independently detrimental effects on CVE is unknown. In this study, we test the hypothesis that in-hospital bleeding (IHB), using Thrombolysis in Myocardial Infarction (TIMI) bleeding definition, would increase the risk of CVE, and CKD might have an additively detrimental effect on CVE in a prospective cohort in an Asia endemic area of kidney disease [9].

## Patients and methods

### Study design

The study was a prospective, national, multicenter, non-interventional, observational design. Patient recruitment and definition of ACS had been previously described in detail [10]. In brief, patients who were aged 20 years or older, who were admitted within 24 h to the hospital with symptoms of ACS and who provided informed consent were eligible to be included in the study. Patient data, such as baseline characteristics, risk factors, clinical presentation, clinical diagnosis, in-hospital interventions as well as medications prescribed were collected from admission to discharge. Patients were followed up at months 3, 6, 9 and 12 post-discharge and data were collected on medication usage, revascularization strategy as well as clinical events, such as death, myocardial infarction (MI), stroke, revascularization and hospitalization. Monitoring for source documentation and accuracy was performed in 5 % of all case report forms at each recruiting site. This study was carried out in accordance with the local regulatory guidelines and international guidelines for Good Epidemiological Practice [11]. Ethics committee approval was obtained at all trial sites. Written informed consent was given by the patients for their information to be stored in the hospital database and used for research.

### Thrombolysis in Myocardial Infarction (TIMI) bleeding classification

TIMI bleeding classification includes major and minor bleeding. TIMI major bleeding is defined as patients with intracranial hemorrhage or a ≥5 g/dl decrease in hemoglobin concentration or a ≥15 % absolute decrease in hematocrit. If observed with blood loss ≥3 g/dl, decrease in hemoglobin concentration or ≥10 % decrease in hematocrit, or no observed blood loss with ≥4 g/dl decrease in hemoglobin concentration or ≥12 % decrease in hematocrit, it is defined as TIMI minor bleeding [12].

### Calculation of kidney function and definition of CKD

The estimated glomerular filtration rate (GFR) was calculated using the Modification of Diet in Renal Disease (MDRD) Study equation [GFR = 186.3 × (serum creatinine in mg/dl)^−1.154^ × (age)^−0.203^ × (0.742 if female)] [13]. Chronic kidney disease was defined as a GFR <60 ml/min per 1.73 m^2^. This range corresponds to stage 3 or higher CKD by the National Kidney Foundation’s classification scheme and helps identify individuals with clinically significant CKD [14].

### Statistical analysis

The sample size for the Taiwan ACS Full Spectrum Registry was calculated as follows. There are about 50000 new ACS cases per year in Taiwan. Based on a known background incidence rate of 0.0025, a sample of 2395 patients would achieve 80 % power to detect an additional incidence rate of 0.003 with a precision of 0.2 % and 95 % confidence interval. Taking into account a dropout rate of 20 %, a sample of 3000 was considered to be adequately representative.

All data were expressed as mean ± standard deviation (SD). For comparability between groups, a Chi-square test or Fisher’s exact test was used for categorical variables and one-way analysis of variance (ANOVA) was adopted for continuous variables. One-year CVE analysis was performed using Kaplan–Meier survival curves and the log-rank test. Univariate and multivariate logistic regression analyses were conducted to analyze odds ratio (OR) and Cox regression model was used for hazard ratio (HR) calculation for IHB or CVE. The adjusted variables in model 1 include age and sex. The adjusted variables in model 2 include model 1 covariates and medicine at discharge (aspirin, clopidogrel, angiotensin-converting enzyme inhibitor, angiotensin II receptor blocker, beta-blocker and statin). Analyses were conducted as time to first event without double counting of events within analyses involving composite end points.

The primary outcome was the composite CVE of death, non-fatal myocardial infarction and non-fatal stroke at 1 year. The secondary outcome was the CVE of death, non-fatal myocardial infarction, non-fatal stroke, re-hospitalization and revascularization at 1 year. We analyzed the whole, STE-ACS and NSTE-ACS populations separately. Statistical analysis was performed using SAS software version 9.2 (SAS Institute Inc., Cary, NC, USA). All statistical analyses were performed using a level of <0.05 with two-sided testing and this was considered as statistically significant.

## Results

### Clinical characteristics

A total of 3183 eligible consecutive patients were enrolled between October 2008 and January 2010 at 39 medical centers and regional hospitals in Taiwan. Among them, 2819 (88.6 %) subjects with renal parameters and 12 months outcome data were analyzed in this study and 1537 (54.5 %) patients had ST-segment elevation acute coronary syndrome (STE-ACS). The subjects included 2230 men and 589 women (male 79.11 %). Mean age was 62.9 ± 13.5 years.

Overall, 53 (1.88 %) patients had TIMI bleeding including 17 (0.60 %) major and 36 (1.28 %) minor. The TIMI major bleeding included 1 intracranial hemorrhage, 1 coronary artery bypass grafting (CABG)-related bleeding, 8 gastrointestinal (GI) bleeding, 3 genitourinary (GU) bleeding and 5 other location bleeding. The TIMI minor bleeding included 2 vascular access sites bleeding, 5 CABG-related bleeding, 23 GI bleeding, 1 GU bleeding and 4 other location bleeding. Compared with no TIMI bleeding subjects, those with TIMI bleeding were older and thinner, had higher grade of Killip class, lower systolic and diastolic blood pressure (DBP) and MDRD GFR at presentation, and lower percentage of cerebrovascular accident (CVA) (Table 1).

**Table 1:** Baseline characteristics between those with and without in-hospital bleeding

Number (%)/mean (SD)	TIMI bleeding (*n* = 53)	No TIMI bleeding (*n* = 2766)	*p* value	IHB(−)/CKD(−) (*n* = 1,846)	IHB(+)/CKD(−) (*n* = 24)	IHB(−)/CKD(+) (*n* = 920)	IHB(+)/CKD(+) (*n* = 29)	*p* value
Sex (male)	38 (71.70 %)	2192 (79.25 %)	0.181	1530 (82.88 %)	21 (87.50 %)	662 (71.96 %)	17 (58.62 %)	<0.01
Age (year)	68.29 ± 13.83	62.77 ± 13.51	<0.01	59.45 ± 13.03	63.30 ± 15.69	69.43 ± 11.93	72.42 ± 10.69	<0.01
Killip								
Class 1	12 (26.67 %)	1394 (62.20 %)	<0.01	1053 (69.23 %)	6 (31.58 %)	341 (47.36 %)	6 (23.08 %)	<0.01
Class 2	8 (17.78 %)	394 (17.58 %)		262 (17.23 %)	5 (26.32 %)	132 (18.33 %)	3 (11.54 %)	
Class 3	7 (15.56 %)	238 (10.62 %)		111 (7.30 %)	2 (10.53 %)	127 (17.64 %)	5 (19.23 %)	
Class 4	18 (40.00 %)	215 (9.59 %)		95 (6.25 %)	6 (31.58 %)	120 (16.67 %)	12 (46.15 %)	
Blood pressure								
SBP (mmHg)	126.39 ± 35.66	139.57 ± 32.63	<0.01	139.64 ± 30.47	126.38 ± 32.98	139.44 ± 36.63	126.41 ± 38.51	0.043
DBP (mmHg)	76.06 ± 21.67	82.10 ± 20.82	0.040	83.63 ± 19.73	73.08 ± 16.82	79.04 ± 22.55	78.70 ± 25.25	<0.01
Heart rate (beat/min)	85.33 ± 30.80	82.03 ± 22.13	0.292	79.79 ± 19.66	84.29 ± 29.36	86.54 ± 25.84	86.21 ± 32.50	<0.01
Height (cm)	162.13 ± 7.93	164.03 ± 7.87	0.082	164.60 ± 7.67	164.67 ± 6.08	162.90 ± 8.15	160.03 ± 8.73	<0.01
Weight (kg)	63.98 ± 12.60	68.70 ± 12.81	<0.01	69.92 ± 12.81	66.27 ± 13.16	66.26 ± 12.47	62.09 ± 12.01	<0.01
Waist circumference	86.47 ± 11.61	90.53 ± 9.51	0.084	90.68 ± 9.23	83.67 ± 13.41	90.19 ± 10.13	88.00 ± 10.88	0.241
Creatinine (mg/dl)	1.73 ± 1.43	1.64 ± 1.82	0.730	0.96 ± 0.19	1.07 ± 0.16	3.01 ± 2.65	2.27 ± 1.76	<0.01
MDRD eGFR	56.05 ± 23.92	73.62 ± 50.90	0.012	92.30 ± 51.68	77.66 ± 11.75	36.14 ± 18.00	38.17 ± 14.77	<0.01
Dyslipidemia	20 (37.74 %)	1073 (39.13 %)	0.837	708 (38.69 %)	9 (37.50 %)	365 (40.02 %)	11 (37.93 %)	0.919
Hypertension	35 (66.04 %)	1741 (63.52 %)	0.706	1040 (56.83 %)	13 (54.17 %)	701 (76.95 %)	22 (75.86 %)	<0.01
Diabetes	24 (45.28 %)	988 (35.91 %)	0.160	505 (27.49 %)	8 (33.33 %)	483 (52.84 %)	16 (55.17 %)	<0.01
Smoker								
Current	22 (41.51 %)	1161 (42.72 %)	0.916	899 (49.48 %)	15 (62.50 %)	262 (29.08 %)	7 (24.14 %)	<0.01
Former	10 (18.87 %)	454 (16.70 %)		264 (14.53 %)	3 (12.50 %)	190 (21.09 %)	7 (24.14 %)	
Never	21 (39.62 %)	1103 (40.58 %)		654 (35.99 %)	6 (25.00 %)	449 (49.83 %)	15 (51.72 %)	
FH of premature CAD	7 (16.67 %)	478 (22.58 %)	0.363	378 (25.93 %)	5 (27.78 %)	100 (15.17 %)	2 (8.33 %)	<0.01
Previous CAD	12 (22.64 %)	663 (23.97 %)	0.822	354 (19.18 %)	4 (16.67 %)	309 (33.59 %)	8 (27.59 %)	<0.01
Previous heart failure	4 (7.55 %)	144 (5.21 %)	0.449	53 (2.87 %)	1 (4.17 %)	91 (9.89 %)	3 (10.34 %)	<0.01
Old CVA	0 (0.00 %)	252 (9.11 %)	0.021	118 (6.39 %)	0 (0.00 %)	134 (14.57 %)	0 (0.00 %)	<0.01
In-hospital medication								
Aspirin	44 (83.02 %)	2551 (92.23 %)	0.014	1738 (94.15 %)	19 (79.17 %)	813 (88.37 %)	25 (86.21 %)	<0.01
Clopidogrel	46 (86.79 %)	2614 (94.50 %)	0.016	1769 (95.83 %)	21 (87.50 %)	845 (91.85 %)	25 (86.21 %)	<0.01
Ticlopidine	0 (0.00 %)	21 (0.76 %)	0.524	13 (0.70 %)	0 (0.00 %)	8 (0.87 %)	0 (0.00 %)	0.889
Warfarin	0 (0.00 %)	27 (0.98 %)	0.470	14 (0.76 %)	0 (0.00 %)	13 (1.41 %)	0 (0.00 %)	0.348
Glycoprotein IIb/IIIa	17 (32.08 %)	457 (16.52 %)	<0.01	317 (17.17 %)	9 (37.50 %)	140 (15.22 %)	8 (27.59 %)	<0.01
Unfractional heparin	29 (54.72 %)	2024 (73.17 %)	<0.01	1361 (73.73 %)	13 (54.17 %)	663 (72.07 %)	16 (55.17 %)	0.020
LMWH	16 (30.19 %)	816 (29.50 %)	0.913	559 (30.28 %)	9 (37.50 %)	257 (27.93 %)	7 (24.14 %)	0.429
ACEI	24 (45.28 %)	1392 (50.33 %)	0.467	1005 (54.44 %)	10 (41.67 %)	387 (42.07 %)	14 (48.28 %)	<0.01
ARB	5 (9.43 %)	316 (11.42 %)	0.651	178 (9.64 %)	1 (4.17 %)	138 (15.00 %)	4 (13.79 %)	<0.01
β-blocker	15 (28.30 %)	1268 (45.84 %)	0.011	873 (47.29 %)	8 (33.33 %)	395 (42.93 %)	7 (24.14 %)	<0.01
Statin	18 (33.96 %)	1367 (49.42 %)	0.026	953 (51.63 %)	9 (37.50 %)	414 (45.00 %)	9 (31.03 %)	<0.01

*TIMI* thrombolysis in myocardial infarction, *SBP* systolic blood pressure, *DBP* diastolic blood pressure, *MDRD* Modification of Diet in Renal Disease Study, *eGFR* estimated glomerular filtration rate, *FH* family history, *CAD* coronary artery disease, *CVA* cerebrovascular accident, *LMWH* low molecular weight heparin, *ACEI* angiotensin-converting enzyme inhibitor, *ARB* angiotensin II receptor blocker

Baseline creatinine was 3.0 ± 2.6 and 1.0 ± 0.2 mg/dl in the CKD (*n* = 949) and non-CKD (*n* = 1870) groups. Compared with non-CKD subjects, those with CKD were older, shorter and thinner, included more women, had higher grade of Killip class, lower DBP and faster heart rate at presentation. They also had more comorbidity including hypertension, diabetes, previous coronary artery disease (CAD), previous CVA and previous heart failure, but lower percentage of smoking and family history of CAD.

### Pharmacological management during admission

Medications prescribed during admission are shown in Table 1. Aspirin, clopidogrel, β-blocker, statins and unfractional heparin were prescribed less often during admission in patients with than those without TIMI bleeding. Glycoprotein IIb/IIa was prescribed more often to TIMI bleeding patients during admission. There was no significant difference regarding use of low molecular weight heparin, warfarin, ticlopidine and renin angiotensin system blockers between two group.

Binary regression analysis found age [OR 1.03, 95 % confidence interval (CI) 1.01–1.06, *p* = 0.015], Killip class (*p* < 0.01), use of glycoprotein IIb/IIIa (OR 2.49, 95 % CI 1.27–4.88, *p* < 0.01) and unfractional heparin (OR 0.36, 95 % CI 0.19–0.68, *p* < 0.01) to be independent predictors for occurrence of IHB (Table 2).

**Table 2:** Predictors for in-hospital TIMI bleeding in binary logistic regression analysis

	Unadjusted OR (95 % CI)	*p* value	Adjusted OR (95 % CI)	*p* value
Age (year)	1.03 (1.01–1.05)	<0.01	1.03 (1.01–1.06)	0.015
Killip IV	1	–	1	
III	0.35 (0.14–0.86)	0.022	0.33 (0.13–0.83)	0.018
II	0.24 (0.10–0.57)	<0.01	0.25 (0.11–0.60)	<0.01
I	0.10 (0.05–0.22)	<0.01	0.11 (0.05–0.24)	<0.01
SBP	0.99 (0.98–1.00)	<0.01		
DBP	0.99 (0.97–1.00)	0.040		
Weight (kg)	0.97 (0.95–0.99)	<0.01		
MDRD eGFR	0.98 (0.98–0.99)	<0.01		
Aspirin	0.41 (0.20–0.86)	0.017		
Clopidogrel	0.38 (0.17–0.86)	0.020		
Glycoprotein IIb/IIIa	2.39 (1.33–4.29)	<0.01	2.49 (1.27–4.88)	<0.01
Unfractional heparin	0.44 (0.26–0.77)	<0.01	0.36 (0.19–0.68)	<0.01
β-blocker	0.47 (0.26–0.85)	0.013		
Statin	0.53 (0.30–0.93)	0.028		

*TIMI* Thrombolysis in Myocardial Infarction, *SBP* systolic blood pressure, *DBP* diastolic blood pressure, *MDRD* Modification of Diet in Renal Disease Study, *eGFR* estimated glomerular filtration rate

### Cardiovascular outcomes

During admission patients with TIMI bleeding had more death and stroke (8.77 vs 1.50 % and 3.51 vs 0.36 %, both *p* < 0.01), but similar recurrent myocardial infarction (1.75 vs 0.75 %, *p* = 0.388) compared with no TIMI bleeding subjects (Table 3). Those with TIMI bleeding still had more death rate at 3, 6, 9 and 12 months follow-up (all *p* < 0.01). The re-hospitalization rate was higher during the 3 and 6 months follow-up in those suffering from TIMI bleeding (both *p* < 0.01).

**Table 3:** Cumulative cardiovascular events during index hospitalization, at 3, 6, 9 and 12 months follow-up

Parameters number (%)/mean ± SD	TIMI bleeding (*n* = 53) (%)	No TIMI bleeding (*n* = 2766) (%)	All (*n* = 2819) (%)	*p* value
In-hospital				
Death	5 (8.77)	46 (1.50)	51 (1.63)	<0.01
Re-infarction	1 (1.75)	23 (0.75)	24 (0.77)	0.388
Stroke	2 (3.51)	11 (0.36)	13 (0.42)	<0.01
3-month follow-up				
Death	8 (15.69)	84 (3.18)	92 (3.42)	<0.01
Myocardial Infarction	2 (.44)	34 (1.31)	36 (1.37)	0.073
Stroke	1 (2.22)	14 (0.54)	15 (0.57)	0.137
Re-hospitalization	14 (31.11)	467 (18.04)	481 (18.27)	0.025
Cardiac	9 (64.29)	311 (67.90)	320 (67.80)	
Non-cardiac	5 (35.71)	144 (31.44)	149 (31.57)	
Both	0 (0.00)	3 (0.66)	3 (0.64)	
Unknown	0	9	9	
Repeat revascularization	1 (2.22)	55 (2.13)	56 (2.13)	0.965
6-month follow-up				
Death	12 (25.53)	117 (4.65)	129 (5.03)	<0.01
Myocardial Infarction	2 (5.13)	59 (2.44)	61 (2.48)	0.284
Stroke	1 (2.63)	24 (0.99)	25 (1.02)	0.318
Re-hospitalization	20 (47.62)	708 (28.51)	728 (28.83)	<0.01
Cardiac	13 (65.00)	483 (69.20)	496 (69.08)	
Non-cardiac	7 (35.00)	192 (27.51)	199 (27.72)	
Both	0 (0.00)	23 (3.30)	23 (3.20)	
Unknown	0	10	10	
Repeat revascularization	2 (5.26)	89 (3.68)	91 (3.71)	0.609
9-month follow-up				
Death	13 (28.26)	135 (5.55)	148 (5.97)	<0.01
Myocardial Infarction	2 (5.71)	76 (3.28)	78 (3.32)	0.425
Stroke	1 (3.03)	30 (1.30)	31 (1.32)	0.387
Re-hospitalization	21 (51.22)	906 (37.59)	927 (37.82)	0.074
Cardiac	14 (66.67)	619 (69.08)	633 (69.03)	
Non-cardiac	6 (28.57)	217 (24.22)	223 (24.32)	
Both	1 (4.76)	60 (6.70)	61 (6.65)	
Unknown	0	10	10	
Repeat revascularization	4 (12.12)	128 (5.54)	132 (5.64)	0.104
12-month follow-up				
Death	15 (31.91)	156 (6.54)	171 (7.03)	<0.01
Myocardial Infarction	2 (5.71)	85 (3.77)	87 (3.80)	0.550
Stroke	1 (3.03)	34 (1.51)	35 (1.53)	0.481
Re-hospitalization	22 (52.38)	1011 (42.50)	1033 (42.67)	0.199
Cardiac	14 (63.64)	675 (67.43)	689 (67.35)	
Non-cardiac	7 (31.82)	236 (23.58)	243 (23.75)	
Both	1 (4.55)	90 (8.99)	91 (8.90)	
Unknown	0	10	10	
Repeat revascularization	4 (11.76)	156 (6.94)	160 (7.01)	0.274

*TIMI* Thrombolysis in Myocardial Infarction

The unadjusted HR of the presence of TIMI bleeding in the whole, STE-ACS and non-ST-segment elevation ACS (NSTE-ACS) populations were 3.66 (95 % CI 2.18–6.1), 2.88 (95 % CI 1.35–6.18) and 5.36 (95 % CI 2.62–10.95), respectively, for the primary end point. For the secondary end point the HR of the presence of TIMI bleeding in the whole, STE-ACS and NSTE-ACS populations were1.74 (95 % CI 1.19–2.53), 1.35 (95 % CI 0.81–2.25) and 2.58 (95 % CI 1.49–4.49), respectively. The association was statistically significant after adjusting for age, sex and medication at discharge in the NSTE-ACS population for the primary outcomes (HR 2.74, 95 % CI 1.29–5.84, *p* < 0.01), but not in the STE-ACS population. For the secondary outcome, TIMI bleeding is still a predictor only in the NSTE-ACS population after adjusting for age, sex and medication at discharge (HR 1.95, 95 % CI 1.10–3.45, *p* = 0.022). There is a trend for in-hospital bleeding being a predictor for the primary end point in those with NSTE-ACS after adjusting for age, sex, medication at discharge, creatinine, weight and Killip class (HR 2.34, 95 % CI 0.94–5.86, *p* = 0.068) (Table 4).

**Table 4:** Multivariable-adjusted odds ratios for the association between in-hospital bleeding and 12 months cardiovascular events

Outcome/hazard ratio (95 % CI)	Unadjusted	Model I	Model II	Model III
Primary outcome				
Overall cohort	3.66(2.18–6.17)*	2.85(1.69–4.81)*	1.57(0.91–2.70)	1.42(0.79–2.58)
STEMI	2.88(1.35–6.18)*	2.25(1.05–4.84)*	0.98(0.44–2.18)	0.96(0.42–2.17)
NSTE-ACS	5.36(2.62–10.95)*	3.82(1.87–7.83)*	2.74(1.29–5.84)*	2.34(0.94–5.86)
Secondary outcome				
Overall cohort	1.74(1.19–2.53)*	1.63(1.12–2.37)*	1.43(0.97–2.10)	1.17(0.75–1.81)
STEMI subpopulation	1.35(0.81–2.25)	1.27(0.76–2.12)	1.16(0.69–1.97)	1.02(0.58–1.80)
NSTE-ACS	2.58(1.49–4.49)*	2.33(1.344.05)*	1.95(1.10–3.45)*	1.46(0.73–2.92)

Model 1: adjusted for age and sex. Model 2: adjusted for Model 1 covariates + medicine at discharge (aspirin, clopidogrel, angiotensin-converting enzyme inhibitor, angiotensin II receptor blocker, beta-blocker and statin). Model 3: adjusted for Model 2 covariates + creatinine, weight and Killip class

* *p* < 0.05

### Influence of TIMI bleeding and CKD on cardiovascular outcome

**Fig. 1:**
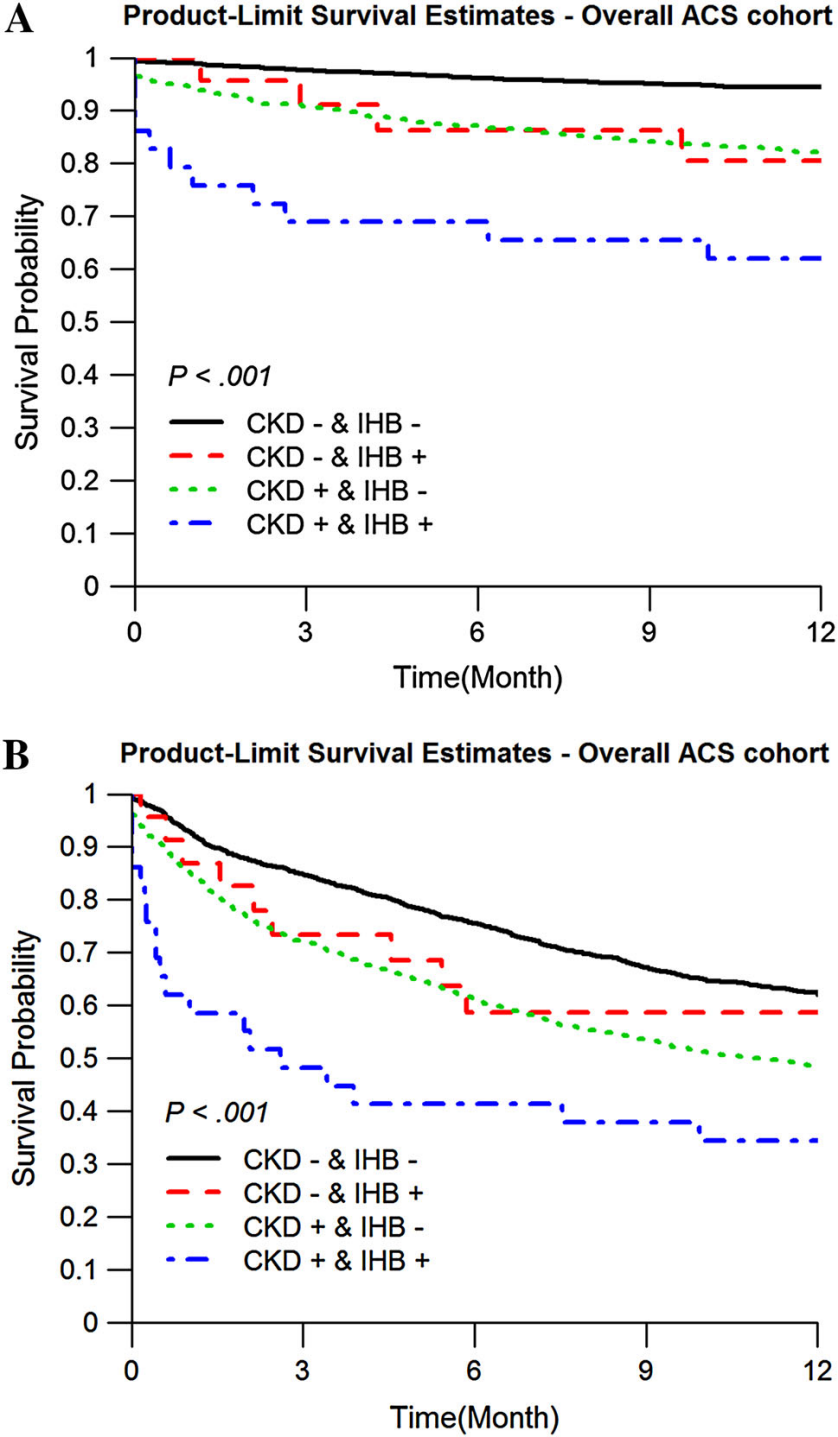
Kaplan–Meier curve analysis for in-hospital TIMI bleeding and CKD on the **a** primary and **b** secondary end points among the whole populations. *TIMI* thrombolysis in myocardial infarction, *CKD* chronic kidney disease, *ACS* acute coronary syndrome

CKD is independently associated with a significant increase of primary end point after adjusting for age, sex and medication at discharge (OR 2.17, 95 % CI 1.63–2.87, *p* < 0.01). The Kaplan–Meier curves show significantly higher primary end point rates among those with IHB and CKD in the whole, STE-ACS and NSTE-ACS populations during 12 months follow-up (all *p* < 0.01) (Fig. 1). We found an additively detrimental effect on the CVE between TIMI bleeding and CKD on the occurrence of primary end point (Table 5). In patients without CKD, TIMI bleeding had a 1.88-fold risk to have primary end point (HR 1.88, 95 % CI 0.68–5.21; *p* = 0.227). When patients had no TIMI bleeding, presence of CKD was associated with a 2.13-fold risk of primary endpoint (HR 2.13, 95 % CI 1.62–2.79; *p* < 0.01), but CKD patients with TIMI bleeding had a 2.98-fold risk for primary endpoint (HR 2.98, 95 % CI 1.55–5.75; *p* < 0.01), compared to the patients without TIMI bleeding and CKD.

**Table 5:** Association between in-hospital bleeding and CKD on primary end point

Groups *n* (%)	Primary end point (+) (*n* = 274) (%)	Primary end point (−) (*n* = 2545) (%)	Adjusted HR (95 % CI)^a^	*p* value
IHB(−)/CKD (−)	101 (36.86)	1745 (68.57)	1	–
IHB(+)/CKD (−)	5 (1.82)	19 (0.75)	1.88(0.68–5.21)	0.227
IHB(-)/CKD (+)	157 (57.30)	763 (29.98)	2.13(1.62–2.79)	<0.01
IHB(+)/CKD (+)	11 (4.01)	18 (0.71)	2.98(1.55–5.75)	<0.01

*IHB* in-hospital bleeding, *CKD* chronic kidney disease

^a^Adjusted for age, sex and medicine at discharge (aspirin, clopidogrel, angiotensin-converting enzyme inhibitor, angiotensin II receptor blocker, beta-blocker and statin)

## Discussion

There are three major findings in this ACS cohort study. First, patients with IHB had higher risk of in-hospital and 12 months death. Second, IHB is associated with poor cardiovascular outcome, especially in those in the NSTE-ACS population. Third, patients with both IHB and CKD had the worst prognosis during the 12 months follow-up. Furthermore, they had additively detrimental effect on the cardiovascular outcome.

By using TIMI bleeding definition our study found that ACS patients with IHB had higher risk of in-hospital and 12 months death. Among the different bleeding definitions, TIMI is more capable than ACUITY in identifying patients with bleeding at higher risk for early mortality [15]. However, the other study suggests that bleeding assessed with clinical criteria by Global Use of Strategies to Open Occluded Coronary Arteries (GUSTO) bleeding criteria is more important than that assessed by laboratory criteria with TIMI bleeding criteria in terms of outcomes [16]. Recently, a consensus report from the Bleeding Academic Research Consortium (BARC) proposed standardized bleeding definitions through the use of a hierarchical approach of describing bleeding severity grade in patients receiving antithrombotic therapy [17]. One study had validated a close association between bleeding events defined according to BARC and 1-year mortality after PCI [18]. More studies might be needed to use BARC bleeding definition to clarify the risk of bleeding among different clinical situations.

Several factors have been reported with IHB such as age, female sex, use of anticoagulation and antiplatelet agents. Different bleeding scores were also developed to calculate the risk of IHB. Mehran et al. used 6 baseline predictors (female sex, age, serum creatinine and white blood cell count, anemia, non-ST-segment elevation MI or ST-segment elevation MI) and 1 treatment-related variable (use of heparin + a glycoprotein IIb/IIIa inhibitor rather than bivalirudin alone) to develop a risk score with c-statistic value 0.74. Similar to the GUSTO IV-ACS study, our study found IHB were related with glycoprotein IIb/IIIa inhibitor administration. Because there was no definite cardiovascular benefit with adding glycoprotein IIb/IIIa to the standard treatment regimen in Taiwan, we used glycoprotein IIb/IIIa inhibitor limited to the very high cardiovascular risk population [19, 20]. Our study also found that higher Killip class was related to IHB. The association might just reflect the disease severity and co-morbidity.

In-hospital bleeding is associated with short-, intermediate-, and long-term mortality among patients hospitalized for ACS and PCI [3, 4, 21]. Patients with IHB after primary PCI in STE-ACS have significantly increased 3-year rates of morbidity and mortality [22]. The deleterious effect of major bleeding was observed within 1 month, between 1 month and 1 year, and between 1 and 3 years. In patients with NSTE-ACS cumulative mortality was also higher in those who had bleeding vs those without at 30 days, 1 year and 3 years [21]. In our study ACS patients with TIMI bleeding had higher in-hospital and 1-year mortality. Although its causal relationship with mortality is unclear, IHB likely identifies patients with an underlying risk for mortality.

Taiwan has been recognized as an endemic area of kidney disease with the highest incidence and prevalence rates of ESRD in the world [9]. Because patients with CKD have more comorbidity, their treatment strategy in ACS is more complicated in the CKD endemic area. As shown in our study CKD is a poor prognosis factor for those with ACS, possibly because of more extensive and severe atherosclerosis coronary tree with plaque composed of greater necrotic core and less fibrous tissue in the CKD than non-CKD subjects [23–25]. Furthermore, poor antiplatelet responsiveness, underuse of reperfusion therapy, fear of contrast-induced nephropathy during coronary procedure and fewer guideline-recommended treatments prescribed may partly explain why the CKD population had poor prognosis in ACS [26–28].

Renal function impairment is associated with platelet dysfunction and coagulopathy and therefore plays an important role in the risk of bleeding. Creatinine only can be integrated as one risk factor in a clinical score which could identify patients at increased risk for bleeding and subsequent 1-year mortality [29]. Estimated GFR and CKD stages were also related to in-hospital bleeding, cardiovascular events and death [30, 31]. Bleeding itself might further cause renal function deterioration and therefore a vicious cycle develops. In this study we first found in-hospital bleeding and CKD might have additively detrimental effect on the cardiovascular outcome. Strategies, such as transradial approach, use of appropriate anticoagulation, antiplatelet therapy and selected use of glycoprotein IIb/IIIa in the high-risk population, to reduce CKD and bleeding are mandatory to reduce subsequent cardiovascular events [32–34].

This study has six main limitations. Firstly, it is a nonrandomized and observational study. Nonetheless, this study provides valuable real-world data on the current practices across the full spectrum of ACS in a CKD endemic area, which could help to improve the ACS management in the CKD population. Second, the mechanism why in-hospital bleeding and CKD have additively detrimental effect of the cardiovascular outcome is unclear. The casual relationship and which one happened first are unknown. Third, the renal end point is not routinely collected after discharge in this registry. Whether those with TIMI bleeding had poor renal outcome is unknown. Fourth, the renal parameter was incorporated into the CRUSADE bleeding risk score, which might be a better score to define bleeding risk. However, we cannot calculate the CRUSADE score because hematocrit was not collected in this registry. Fifth, the prognostic difference between the in-hospital hemoglobin changes and TIMI IHB might provide different insight in clinical judgment. However, serial hemoglobin data were not collected during admission in this registry. Sixth, because the interaction test between ACS type and bleeding on outcomes is non-significant, the finding of excess risk in NSTE-ACS was only hypothesis generating.

## Conclusion

In this real-world registry, we found that patients with IHB had higher risk of in-hospital and 12 months death in the ACS population. Furthermore, patients with both IHB and CKD had the worst prognosis during the 12 months follow-up. Thus, all measures decreasing IHB and preventing CKD in ACS patients are important for eventual cardiovascular risk reduction.

## Appendix: ACS Full Spectrum Registry principal investigators

Kuan-Chen Chang, China University Medical Hospital; Chia-Lin Chao, Taoyuan General Hospital, Department of Health; Yi-Jen Chen, Wan-Fang Hospital; Chien-Cheng Chen, Show Chwan Memorial Hospital; Cheng-Yun Chen, Chia-Yi Christian Hospital; Chung-Yin Chen, Kuang Tien General Hospital; Fu-Tien Chiang, National Taiwan University Hospital; Shao-Yueh Chiang, Cheng Ching Hospital; Li-Ping Chou, Sin Lau Hospital The Presbyterian Church of Taiwan; Ching-Chang Feng, Tainan Municipal Hospital; Charles Jia-Yin Hou, Mackay Memorial Hospital; Kwan-Li Hsu, E-Da Hospital; Tsuei-Yuan Huang, Chi-Mei Hospital; Gwo-Ping Jong, Taichung Armed Forces General Hospital; Yu-Lin Ko, Taipei Tzu Chi General Hospital; Wen-Ter Lai, Kaohsiung Medical University Chung-Ho Memorial Hospital; Wen-Lieng Lee, Taichung Veterans General Hospital; Chun-I Lee, Pingtung Christian Hospital; Meng-Huan Lei, Lo-Tung Po-Ai Hospital; Ai-Hsien Li, Far Eastern Memorial Hospital; Yi-Heng Li, National Cheng Kung University Hospital; Jou-Wei Lin, National Taiwan University Hospital, Yun-lin Branch; Tin-Kwang Lin, Dalin Tzuchi General Hospital; Jih-Min Lin, Kee-lung Hospital, Department of Health; Shing-Jong Lin, Taipei Veterans General Hospital; Hung-Shun Lo, Cathay General Hospital; Guang-Yuan Mar, Kaohsiung Veterans General Hospital; Chun-Ming Shih, Taipei Medical University Hospital; Kou-Gi Shyu, Shin Kong Wu Ho-Su Memorial Hospital; Cheng-Dao Tsai, Changhua Christian Hospital; Chuen-Den Tseng, National Taiwan University Hospital; Kwo-Chang Ueng, Chung Shan Medical University Hospital; Ji-Hung Wang, Hualien Tzu Chi General Hospital; Kuang-Te Wang, Mackay Memorial Hospital, Taitung Branch; Ming-Shien Wen, Linkou Chang Gung Memorial Hospital; Szu-Chi Wen, Hsin Chu General Hospital, Department of Health; Chiung-Jen Wu, Kaohsiung Chang Gung Memorial Hospital; Shih-Peng Yang, Tri-Service General Hospital; Wei-Hsian Yin, Cheng-Hsin Hospital.

## Abbreviations

CVD: Cardiovascular disease
ACS: Acute coronary syndrome
CVE: Cardiovascular events
PCI: Percutaneous coronary intervention
CKD: Chronic kidney disease
IHB: In-hospital bleeding
TIMI: Thrombolysis in myocardial infarction
MI: Myocardial infarction
GFR: Glomerular filtration rate
ESRD: End-stage renal disease
MDRD: Modification of diet in renal disease
STE-ACS: ST-elevation acute coronary syndrome
DBP: Diastolic blood pressure
CAD: Coronary artery disease
CVA: Cerebrovascular accident
NSTE-ACS: Non-ST-elevation acute coronary syndrome
